# Key Challenges in Screening Blood Donors for Hepatitis B Virus

**DOI:** 10.3390/v18010023

**Published:** 2025-12-23

**Authors:** Maha A. Badawi, Sahar Eldakhakhny, Mohamed Ali, Mazen Badawi, Jaffar Khiariy, Yagoub Bin-Taleb, Salwa I. Hindawi

**Affiliations:** 1 Hematology Department, Faculty of Medicine, King Abdulaziz University, Jeddah 21589, Saudi Arabia; 2 Hematology Research Unit, King Fahd Medical Research Center, King Abdulaziz University, Jeddah 21589, Saudi Arabia; 3 Blood Transfusion Services Unit, King Abdulaziz University Hospital, Jeddah 21589, Saudi Arabia; 4 Diagnostic Virology Laboratory, King Abdulaziz University Hospital, Jeddah 21589, Saudi Arabia; 5 Abbott Laboratories GmbH, Dubai P.O. Box 32002, United Arab Emirates; 6 Infectious Diseases Division, Internal Medicine Department, King Faisal Specialist Hospital and Research Centre, Jeddah 21499, Saudi Arabia; 7 Department of Pathology & Laboratory Medicine, King Faisal Specialist Hospital and Research Centre, Jeddah 21499, Saudi Arabia; 8 Pediatric Gastroenterology Unit, Pediatric Department, King Abdulaziz University Hospital, Jeddah 21589, Saudi Arabia; 9 Saudi Society of Transfusion Medicine & Services, Jeddah 21589, Saudi Arabia

**Keywords:** blood donation, donor screening, hepatitis B virus

## Abstract

Technological advancements in blood donor screening have significantly improved blood safety. However, certain testing challenges and limitations continue to face blood banks in donor screening for the hepatitis B virus, resulting in occasional cases of transfusion transmission. These cases are mostly related to donors presenting within the window period and donors with occult hepatitis B infection. There are several other challenges that professionals in transfusion medicine, infectious diseases, gastroenterology, and public health must be aware of. Maintaining the highest test sensitivity is a key parameter for enhancing blood safety, and the review describes current recommendations in this regard, along with relevant advancements. The diversity of viral genotypes and the potential for mutations affecting the surface antigen may negatively affect the performance of both serologic and nucleic acid tests. Serologic tests may also be affected by several interferences, endogenous or exogenous to the sample. A clear understanding of these challenges is necessary to create effective policies and procedures and to properly manage atypical cases.

## 1. Introduction

Despite significant advances in blood donor screening for the prevention of transfusion-transmitted infections, hepatitis B virus (HBV) infection remains a threat to the safety of blood recipients around the globe. The residual risk of HBV transmission from transfusion varies considerably between countries. For example, the residual risk of HBV transmission through blood was reported to be 1 in 7.5 million donations in Canada, 45 in 1 million donations in South Africa, 156 in 1 million donations in Mexico, and 1 in 408 donations in Burkina Faso [1,2,3,4]. This variability is explained by the prevalence of HBV in the population and by the donor screening methods utilized by blood collection agencies [5]. Many low-income countries rely on rapid testing kits for HBV surface antigen (HBsAg), while most middle- and high-income countries utilize immunoassay (serological) testing for HBsAg combined with testing for the core (HBcAb) and surface (HBsAb) antibodies, with or without nucleic acid testing (NAT). Combining serological testing and NAT provides significant protection against the risk of HBV transmission through transfusion; however, the risk can virtually never be eliminated, mostly secondary to donations within the window period and donations by individuals who have occult hepatitis B infection (OBI). Blood banks face these and other challenges, such as false-positive results that may cause unnecessary deferrals and increased donor anxiety. While guides to the interpretation of serological tests for HBV are commonly available through many resources (Table 1), this review provides professionals in transfusion medicine, gastroenterology, infectious diseases, and public health with a general overview of the various challenges that may affect testing results. It will equip professionals in these specialties with the knowledge to understand testing limitations, develop an approach to counsel blood donors, determine eligibility, and recognize the potential need for policy updates. We provide an overview of the structure of HBV, genetic mutations that affect detectability, the window period, OBI, vaccine breakthrough infections, sensitivity of tests, and interferences with testing.

## 2. Hepatitis B Virus Structure

**Figure 1:**
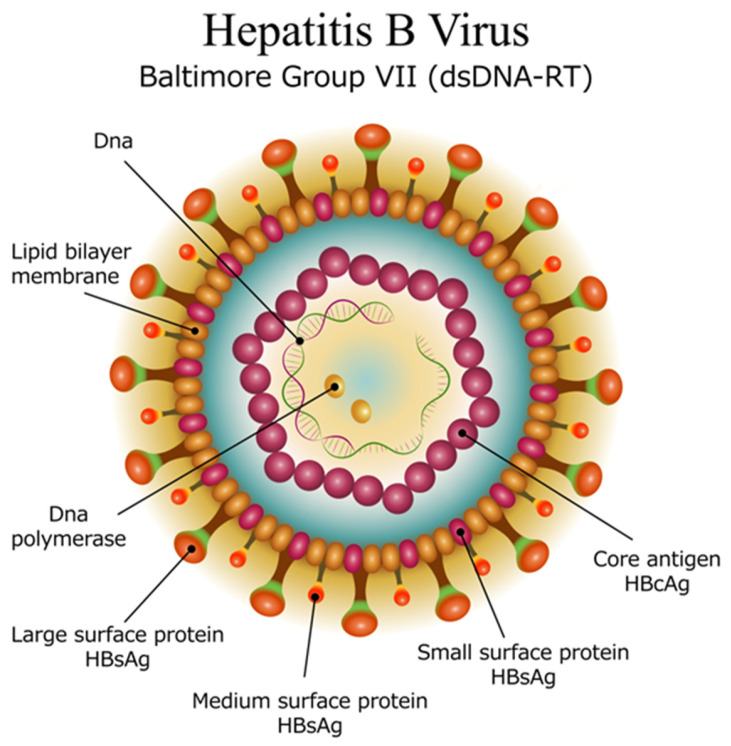
Structure of the Hepatitis B Virus: The lipid bilayered membrane embeds the surface antigen (s antigen), which exists in 3 variants (small, medium, and large). Inside the virus is the icosahedral nucleocapsid made of capsomers (core antigens). Inside the nucleocapsid, the DNA of the virus is present in a circular, partially double-stranded form, along with an attached polymerase. (Image licensed from Shutterstock and modified).

HBV is a small, enveloped virus that belongs to the *Hepadnaviridae* family. The lipid bilayered membrane embeds the surface antigen (s antigen), while additional antigens (pre-S 1 and pre-S 2) are attached to its external surface (Figure 1). Inside the virus is the icosahedral nucleocapsid made of capsomers (core antigens). Inside the nucleocapsid, the DNA of the virus is present in a circular, partially double-stranded form, along with an attached polymerase. The viral genome consists of 4 main regions [4]:

1. The precore (pre-C) and core (C) gene, which encodes the core protein (HBcAg) and the e antigen (HBeAg) involved in immune evasion.
2. The surface (S) gene, which encodes the surface proteins (HBsAg) that form the viral envelope. There are three main surface antigens produced: large (L), middle (M), and small (S), each of which has different roles in the virus’s ability to infect host cells.
3. The polymerase (P) gene, which encodes the viral polymerase responsible for reverse transcription of the viral RNA into DNA during replication.
4. The X Gene, which encodes the X protein (HBx) that plays a role in the regulation of viral replication and the modulation of the host cell environment, potentially contributing to oncogenesis.

Ten genotypes of HBV have been recognized (named A to J). While genotype A is commonest in North America and Northern Europe [8], genotypes B and C are more common in East Asia, and genotype D in the Eastern Mediterranean region [9].

Several techniques are available for HBV genotyping. Among these, sequencing and phylogenetic analysis of the complete HBV genome remain the gold standard for accurate genotype determination [10]. Other commonly used approaches include restriction fragment length polymorphism (RFLP) analysis [11,12], polymerase chain reaction (PCR) with genotype-specific primers and probes [13,14,15], hybridization-based methods [16,17], and enzyme-linked immunosorbent assay (ELISA) techniques [18,19].

HBV genotyping is primarily conducted in clinical and research settings to guide treatment decisions and support epidemiological studies, as different genotypes can show variable responses to antiviral therapies. In blood banks, genotyping is considered an advanced testing approach that typically utilizes PCR with genotype-specific primers to determine the viral genotype. This method is particularly valuable for assessing potential risks and enhancing blood safety by detecting low viral load cases such as occult HBV infections, identifying “window period” infections, and understanding regional genotype prevalence.

However, HBV genotyping is not routinely included in standard blood donor screening protocols. Only a limited number of commercial discriminatory NAT assays have been approved for use, and many of these are designed to detect only the most common genotypes (A, B, C, and D), which may lead to missed detection of less prevalent variants. Therefore, understanding the geographic distribution of HBV genotypes is crucial for optimizing screening strategies, improving detection accuracy, and ensuring the overall safety of the blood supply.

## 3. Hepatitis B Virus Gene Mutations

Mutations in the surface gene of HBV can result in altered or truncated HBsAg, preventing recognition of the virus by antibodies used in standard tests [20]. Structural changes in the HBsAg gene could reduce the expression of the antigen and lead to unstable binding in commercial assays [21]. For instance, mutations at amino acid position 118 significantly affect the sensitivity of ELISA to detect HBsAg [22]. Another HBV variant with a novel insertion in the surface protein leads to hyper glycosylation. This mutation impairs the ability of standard diagnostic assays to detect HBV and hinders its recognition by antibodies induced by vaccination or natural infection. These findings suggest that such mutations can significantly impact the efficacy of current HBV diagnostic methods and immune responses, increasing the risk of undetected infections [23].

Conventional HBsAg assays, including ELISA, and rapid detection devices may fail to detect HBsAg mutants, leading to false-negative results. A study in Pakistan highlighted the inadequacy of these assays in detecting mutant HBsAg alone, with up to 17% detection failure in known HBsAg-positive cases, emphasizing the need for more sensitive and specific testing methods such as PCR [24]. Thirteen commercial assays underwent performance evaluation in France to assess their ability to detect HBsAg variants. The assays detected the variants with overall positive signals ranging from 62.9% to 97.9%, but specific variants were undetectable by at least one assay [25].

Some specific mutations in the S gene region, associated with reduced sensitivity of commercial immunoassays, were identified through full gene sequencing. These include I126T, G145R, C124R, C124Y, K141E, and D144A [26,27].

S gene mutations do not only affect serological testing; they may impair viral particle secretion, consequently reducing the sensitivity of NAT [28]. An analysis of blood donations in Southern China reported samples that were reactive to HBsAg using ELISA but nonreactive using NAT. Out of 100,252 donations, 79 samples were further investigated. The study found that 21.5% of those samples were confirmed to be HBsAg-positive, the majority being of genotype B. Specific mutations in the HBV genome, such as T1719G, were identified [29].

Although not part of current protocols for blood donor screening, DNA sequencing may be useful when evaluating donors with discordant results in HBsAg testing and NAT to identify viral genotype and potential mutations.

## 4. The Window Period

The diagnostic window period is defined as the phase elapsing between the time of infection and the time of detection of the viral marker by the screening assay. The length of the diagnostic window for a particular assay depends on the marker, the screening assay category, the sensitivity of the assay used, and the replication kinetics of the virus during the early infection period [30].

**Figure 2:**
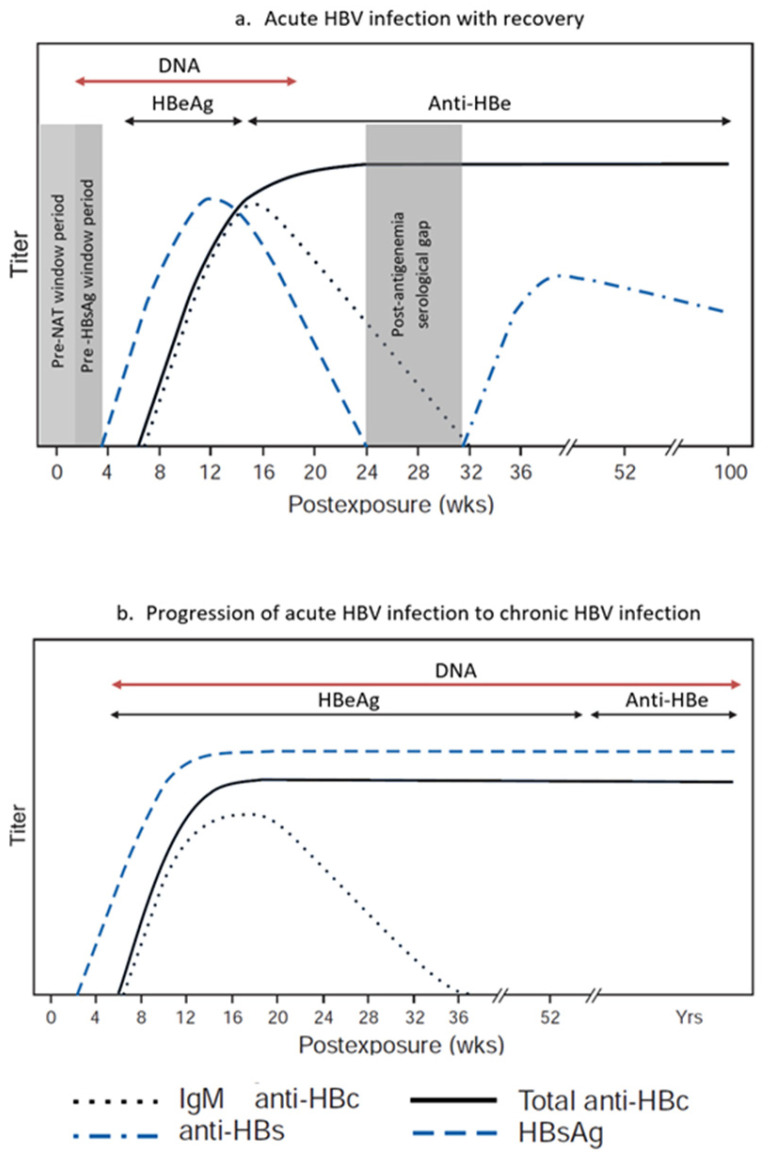
The roles of serology and molecular testing in the evaluation of Hepatitis B virus. infection stages. In acute HBV infection, after the window period, the first detectable viral marker is DNA, followed by Hepatitis B surface antigen (HBsAg). In cases of recovery, a serological gap of a few weeks exists between HBsAg resolution until the appearance of Hepatitis B surface antibody (HBsAb). During that gap, Hepatitis B core antibody may be the only positive marker. In cases of progression to chronic infection, HBsAb may not be detectable, but residual viremia may be detected through nucleic acid testing.

Among all tests used for blood donor screening for HBV, NAT assays have the shortest window period, reported to range from 10 to 20 days after infection (Figure 2) [31]. Depending on the relative sensitivities of the HBsAg and HBV NAT assays used, HBV DNA can be detected 2 to 5 weeks after infection, and up to 40 days (mean 6–15 days) before HBsAg. It is currently well accepted that NAT sensitivity is improved with the use of individual donor NAT (ID-NAT) in comparison with testing in mini-pools (MP-NAT) [32]. NAT screening in blood donations reduces the risk of transfusion-transmitted infections and shortens the window period for serological marker screening. Therefore, a sensitive NAT screening method for HBV and the recruitment of regular low-risk donors are critical for blood safety [33].

The use of HBsAg assays with high analytical sensitivity in addition to NAT-based assay is key for the detection of early infection. In one study of a total of 10,981,776 donations, ID-NAT and serology were found to be complementary in detecting HBV infection in first-time donors, but HBV-DNA was superior to HBsAg detection in repeat donors [34]. NAT yield, defined as the number of donors identified to have the virus only through NAT, is commonly used to estimate residual risk of viral transmission through transfusion in a specific population [35]. Although donors presenting within the NAT window period may not be detected using screening assays, it is essential to attempt to exclude them using a donor history questionnaire that covers the risk factors of HBV in a particular population.

## 5. Analytical Sensitivity of Assays for HBsAg

HBsAg analytical sensitivity refers to an assay’s ability to detect the smallest amount of HBsAg in a sample. High sensitivity is essential for early diagnosis and ensuring the safety of blood donations [36,37]. To detect blood donors with low viremia, the minimum limit of detection for HBsAg assays recommended by the European Union and World Health Organization (WHO) is 0.13 IU/mL [31,38], while it is 0.2 IU/mL according to UK requirements [39].

Traditional HBsAg assays used in donor screening that meet the various standards are effective but may miss low-level infections, including occult HBV infection (OBI) and vaccine breakthrough cases. Ultra-sensitive assays like the Abbott ARCHITECT^®^ HBsAg NEXT, with an analytical sensitivity of 0.005 IU/mL, demonstrate up to 14.5-fold greater sensitivity than conventional assays and have shown the ability to detect HBsAg earlier in seroconversion panels and in DNA-positive/HBsAg-negative samples. Notably, this assay detected 7 of 12 HBV DNA-positive samples missed by standard HBsAg tests, without compromising specificity [40]. Similarly, the Fujirebio Lumipulse-G-HBsAg-Quant assay, with a quantification limit as low 0.0033 IU/mL, identified HBsAg in 25% of previously negative samples from individuals in the window period and with OBI [41]. These findings suggest that ultra-sensitive assays are particularly valuable in identifying OBIs, early-stage infections, and HBV vaccine breakthrough infections which are critical for ensuring blood safety in high-prevalence regions, especially in regions where NAT is not widely available [40,42,43].

The risks associated with reliance on rapid HBsAg testing kits for donor screening stems from its limited sensitivity. A systematic review and meta-analysis published in 2024 evaluated the diagnostic accuracy of various HBsAg assays highlighted significant improvements in their sensitivity and implications for clinical practice. While chemiluminescent microparticle immunoassay had excellent sensitivity, none of the 33 studied rapid diagnostic tests for HBsAg met the limit-of-detection criteria set by the European Union and the WHO [44]. Newer rapid diagnostic tests have become available since, which meet the required limit of detection, but their use for blood donor screening must be further explored [45].

## 6. Interferences

Despite their high sensitivity and specificity, immunoassays are prone to interference by a variety of substances that can be classified into exogenous (process and analyzer-related) and endogenous (sample content-related) factors [46]. Interfering factors may cause false-positive or false-negative results and must be considered in case of discrepant results. Examples of interferences in immunoassays are provided in Table 2.

Heterophilic antibodies are naturally occurring, low-avidity human antibodies targeting nonspecific immunogens. They bridge assay antibodies in noncompetitive immunoassays. On the other hand, human anti-animal antibodies (such as human anti-mouse antibodies) are antibodies against a specific animal species that may be formed after exposure to animal immunogens in a social or therapeutic setting (such as after administration of monoclonal antibodies). Both types of antibodies may result in falsely increased or decreased measurements of analytes, leading to false-positive or false-negative results [47,48]. The effect of these antibodies may be mitigated through the use of neutralizing material, such as nonimmune mouse IgG. Vaccines, common interventions used in the general public including blood donors, have been recognized to affect specific assays. For example, hepatitis B vaccine can result in a transiently positive result for HBsAg testing up to 14 days post vaccination [49]. Influenza vaccine has been reported to be associated with false-positive results for serological testing for HCV, HIV, and HTLV, and there are rare reports of them causing false-positive HBsAg results [50].

Manufacturers usually provide assurance that a specific substance is not expected to provide anomalous results if the substance is present up to specific limits; for example, a reagent manufacturer states that the assay is not affected by icterus (bilirubin < 40 mg/dL), hemolysis (hemoglobin < 2200 mg/dL), lipemia (triglycerides < 2200 mg/dL), and biotin levels below 1200 ng/mL [51]. Further, test results may be false-positive if sample-to-sample carryover occurs. This phenomenon describes the effect of a sample with a high analyte concentration on subsequent samples because of particle carryover. Carryover is greatly reduced through the use of disposable pipette tips [52].

## 7. Limitations of NAT

The introduction of NAT has resulted in significant improvements in blood safety worldwide, mainly through viral detection before seroconversion [53]. Commercial assays such as cobas TaqScreen MPX v2 and Procleix Ultrio Plus achieve 95% limits of detection (LOD) between 2 and 4 IU/mL. These assays have significantly reduced the window period left by HBsAg testing, narrowing it to an estimated eclipse phase of ~15 days [43].

The analytical sensitivity of HBV NAT assays is influenced by several technical factors. Primer-probe mismatches, especially in regions with high HBV genetic diversity (e.g., genotypes B, C, and D), can reduce amplification efficiency and lead to false negatives [43]. Furthermore, nucleic acid loss during extraction and purification is a critical concern. Comparative studies have demonstrated significant variability in DNA recovery across different extraction platforms, with some systems yielding up to twice the amount of viral DNA as others [54]. The choice of extraction reagents and protocols, such as surfactant-based versus magnetic bead-based methods, also affects the limit of detection and overall assay performance [55]. NAT’s performance in detecting low levels of viremia is shown to improve with individual-donor NAT when compared with testing in mini pools [54,56].

False-positive results in HBV NAT may arise from nonspecific amplification, carryover effect, or borderline reactivity near the assay’s detection threshold. These cases necessitate repeat testing and discriminatory assays to confirm infection status. Importantly, some non-repeatable NAT-reactive donations may represent true low-level infections rather than assay artifacts, requiring cautious interpretation [28].

To mitigate these limitations, blood centers are increasingly adopting ID-NAT, serial testing, and integrated screening algorithms that combine NAT with serological markers such as anti-HBc. These strategies enhance detection accuracy and reduce residual risk.

Despite their effectiveness, NAT technologies continue to constitute economic challenges to blood suppliers, limiting the ability to use them in many countries [5].

## 8. Occult Hepatitis B Virus Infection

OBI is defined as the presence of replication-competent HBV DNA (i.e., episomal HBV covalently closed circular DNA [cccDNA]) in the liver and/or HBV DNA in the blood of people found to be negative for HBsAg by currently available assays. The viral suppression in OBI, alongside the low replication level of cccDNA, has been explained by the host’s strong immune suppression of HBV replication and gene expression [57].

Based on the serological profile of hepatitis B antibodies, OBI can be classified as seropositive (Anti-HBc- and/or Anti-HBs-positive) or seronegative (Anti-HBc- and Anti-HBs-negative). The majority of cases of OBI are seropositive [58]. In seropositive OBI, HBsAg may have been undetected after resolving acute hepatitis B in the presence of a chronic infection status. The duration and detection of HBsAg reactivity are highly variable. The serum level of HBV DNA, when detected, in true OBI is usually low (below 200 IU/mL). However, when the level is comparable to those detected in overt HBV infection, this should be considered false OBI. False OBI occurs mainly due to HBsAg escape mutants detected by some HBsAg assays with poor sensitivity to detect the S gene mutant of HBV [59]. Seronegative OBI is estimated to constitute from 1% to 20% of OBI patients, who might not be forming HBV antibodies (HBc and HBs) or have been seronegative since the beginning of the infection [57].

Accurately determining the prevalence of OBI is a real challenge, as it relies on the sensitivity of the testing assays used to test for HBsAg and HBV DNA. Additionally, the prevalence in the population affects the findings. For example, OBI is higher in countries with high HBV prevalence like West Africa and East Asia (1:100–1000) compared with Western Europe, North America, and Australia (below 1:5000); and higher rates are seen in people with existing risk factors like chronic liver disease (which shows an increase of 40–70%) and patients with co-infection with HCV (15–33%) and HIV (10–45%) compared with blood donors and general population rates (less than 0.5%) [57,60,61,62,63,64].

Feasibility of OBI diagnosis depends on the sensitivity of the assays used to detect HBV DNA in liver tissue and/or blood samples [30]. HBsAg assays with poor sensitivity lead to misdiagnosis of HBV infection compared with ultrasensitive assays [36]. Recent studies demonstrate that ultrasensitive HBsAg assays with a limit of detection (LOD) of 5.0 to 5.2 mIU/mL, compared with currently used HBsAg assays (with an LOD of 20–50 mIU/mL) have a sensitivity performance close to mini-pool NAT and enhanced detection of OBI [36].

Furthermore, OBI could be missed through NAT, since the viremia is not consistent. Repeated NAT over time typically yields both positive and negative results. Poorly sensitive HBV DNA assays can also miss OBI, leading to false-negative results, but currently available NAT and real-time PCR-based assays have sufficient performance to detect many OBI cases. Using highly sensitive NAT, investigators from Slovenia have demonstrated that hepatitis B was transmitted from three repeatedly negative HBsAg donors with undetected HBV DNA to nine recipients through blood components [65]. These cases allowed for the revision of the minimal estimated HBV DNA infectious dose to be approximately 3.4 IU/mL instead of 20 IU/mL. Moreover, this estimation needs further reduction to 0.15 IU/mL in order to prevent HBV transmission [66].

Detection of anti-HBc is considered a surrogate marker to detect OBI in blood, organ, and tissue donors and in patients who are about to receive immunosuppressive therapy [67]. Many countries that have low prevalence rates for HBV elect to defer all donors with positive anti-HBc. In other countries, where such a policy could affect blood availability, alternative policies exist, including the determination of donor eligibility based on anti-HBs. Studies from Japan suggest that it is unlikely for blood to transmit HBV if donors are anti-HBc-positive and the level of anti-HBs exceeds 100 mIU/mL (Abbott Architect, Illinois, IL, USA) [68]. As safe anti-HBs levels vary according to the manufacturer, a safety margin is essential; this led Japan to use a level of 200 mIU/mL as a stratification measure to accept or defer donors with anti-HBc.

## 9. Vaccine Breakthrough

Vaccination against HBV is a safe and effective way of prevention; however, this method has a few gaps. Infections in vaccinated individuals (vaccine breakthrough) and vaccine non-responsiveness are occasionally seen. As laboratory findings in these cases may vary from usual patterns, they can present threats to blood safety [69,70].

The recombinant HBV vaccine is considered 95% to 100% effective in preventing chronic HBV infection for at least 30 years following vaccination. Its efficacy can decrease with advanced age and other factors [71]. In order to achieve the best and longest-term effect, the whole vaccine series should be completed starting from infancy. However, it is not uncommon to see immunity to HBV fading. A booster vaccine dose may be necessary in some patients to extend the vaccine’s effectiveness. People who develop an anti-HBs titer of at least 10 IU/L (adequate anti-HBs titer) following the completion of a recommended vaccine schedule are considered protected for life. Exceptions are some immunocompromised persons and patients with chronic renal disease, especially those on dialysis. These groups may require periodic boosters with higher doses, if their anti-HBs titer falls below 10 IU/L, as well as regular follow-ups. On the other hand, in immunocompetent individuals, although anti-HBs titers may become nondetectable over time, immunity memory can persist and remain protective. Higher titers of anti-HBs following the initial immunization are associated with longer antibody persistence.

Vaccine breakthrough infections differ from typical acute HBV infection. In acute infection in unvaccinated individuals, the appearance of viral markers in the peripheral blood follows a consistent pattern and order: HBV DNA followed by HBsAg, HBeAg, and then anti-HBc. Resolution of the typical acute infection is marked by loss of serum HBsAg and HBV DNA and the appearance of anti-HBs. In contrast, during vaccine breakthrough infections, HBV DNA becomes detectable in vaccinated individuals who have protective levels of anti-HBs, but this is also limited by the sensitivity of the testing assays. HBsAg detection may be delayed, transient, or absent, and anti-HBc may be negative [72]. Cases of HBV breakthrough infection have also shown atypical results, including isolated positive anti-HBs [69].

One of the causes of breakthrough infection is the occurrence of HBsAg escape mutants due to mutations in specific epitopes, such as the “a” determinant region of HBsAg. If this occurs, it may allow the virus to escape neutralizing antibodies formed by the vaccine and HBV immunoglobulin. In Taiwan a study found that the prevalence of HBV S mutants in vaccinated HBV DNA-positive children increased from 8% in 1984 to 23% in 1999 [73].

## 10. Conclusions

The complexity of HBV and the multitude of factors affecting the results of various screening assays suggest that blood suppliers need a multi-layered approach combining recognition and deferral of high-risk donors, and utilization of ultra-sensitive HBsAg assays, highly sensitive NAT, and possibly anti-HBc screening. It is essential to adhere to manufacturer instructions regarding the environmental conditions of analyzers, the conditions of reagents, and the proper handling and processing of samples. HBV will continue to pose a threat to the blood supply, but the residual risk varies according to the prevalence of the infection in the donor population and the screening tests used. Pathogen-reduction technologies add layers of safety but are not yet available for all blood components. Healthcare professionals are reminded that transfusion of blood and blood components will continue to carry the inherent risk of transmitting transfusion-transmitted infections. Implementation of patient blood management and hemovigilance systems at the national level is essential to reduce the risk of recipient exposure.

## Figures and Tables

**Table 1:** Interpretation of serological tests for hepatitis B virus infection.

HBsAg	Total Anti-HBc	Anti-HBc IgM	Anti-HBs	HBV DNA	Possible Interpretation
−	−	−	−	−	Never infected; susceptible if never vaccinated or vaccine failure
+	−	−	−	+ or −	Early acute infection (if HBV DNA is positive); transiently positive for HBsAg after vaccination (if HBV DNA is negative)
+	+	+	−	+	Acute infection
−	+	+	+ or −	+ or −	Acute resolving infection; “window period” if anti-HBs is negative
−	+	−	+	−	Recovered from past infection and immune
+	+	−	−	+	Chronic HBV infection
−	−	−	+	−	Immune from vaccination; passive anti-HBs transfer after hepatitis B immune globulin administration
−	+	−	−	+ or −	Isolated total anti-HBc-positive
−	+ or −	−	+ or −	+	Occult HBV infection
+ or −	+	+ or −	+ or −	+	Possible HBsAg mutant infection

Abbreviations: HBsAg = hepatitis B surface antigen, Anti-HBc = hepatitis B core antibody, IgM = immunoglobulin M, Anti-HBs = hepatitis B surface antibody, + = reactive test, − = nonreactive test. Table modified from Center for Disease Control (CDC) sources. It does not imply endorsement by CDC of any specific commercial products, manufacturers, companies, or trademarks. The material is available on the agency website for no charge without the need to obtain copyright permission [6,7].

**Table 2:** Causes of potential interference with immunoassays.

Exogenous	Endogenous
Errors in calibrator, control, or reagent storage or constitution. Undetected bubbles Errors in pipetting or washing	Type 1 (may be detectable in the pre-analytical phase) Hemolysis Bilirubin Lipemia
	Type 2 (not detectable in the pre-analytical phase) Heterophilic antibodies Anti-animal antibodies Biotin Anti-streptavidin antibodies Autoantibodies Cross-reactivity (e.g., drugs) Hook effect (high level of an analyte causing a disproportionately low signal in non-competitive immunoassays). Paraproteins Vaccinations
